# PD97 Comparison Of Clinical Outcomes Of Self-Expandable Versus Balloon-Expandable Valves For Transcatheter Aortic Valve Replacement: A Population-Based Cohort Study

**DOI:** 10.1017/S0266462324003441

**Published:** 2025-01-07

**Authors:** Chinghu Chung, Matt Moore, Ya-Ping Hung, Ju-Yi Hsu

## Abstract

**Introduction:**

Transcatheter aortic valve replacement (TAVR) has become a leading treatment for patients with severe aortic stenosis (AS). Recent studies comparing TAVR outcomes with balloon-expandable valves (BEVs) and self-expandable valves (SEVs) show generally similar results, although BEVs have lower rates of moderate-to-severe aortic regurgitation and pacemaker implantation. This study aimed to compare the clinical outcomes of SEVs and BEVs in Taiwan.

**Methods:**

The Taiwan National Health Insurance Research Database (NHIRD) is a representative claims database capturing 99.9 percent of residents. We identified patients who underwent TAVR with either a SEV or BEV in 2021 using the NHIRD. The outcomes were six-month survival, length of hospital stay (LOS) and intensive care unit (ICU) stay, postoperative complications, and healthcare expenditure. We used inverse probability of treatment weighting (IPTW) based on age, gender, and Charlson Comorbidity Index score to identify the effect of TAVR valve type on LOS and ICU stay, postoperative complications, and healthcare expenditure. Differences between SEVs and BEVs for IPTW-weighted Kaplan-Meier curves of overall survival were measured with the log rank test.

**Results:**

Among the patients identified who underwent TAVR, 366 received a SEV and 132 received a BEV. The mean ages were 82.70 (standard deviation [SD] 8.08) years and 82.25 (SD 7.53) years, respectively. The hazard ratio for six-month mortality rate for SEVs compared with BEVs was 2.78 (95% confidence interval 1.52, 5.09). The six-month mortality rate was also significantly higher for SEVs than for BEVs (13.11% versus 4.55%). For clinical outcomes, the mean total LOS (14.78 [SD 12.19] versus 14.45 [SD 12.96] days), mean ICU stay (5.91 [SD 9.78] versus 6.23 [SD 8.04] days), rate of complications (<3%) and in-hospital healthcare costs (USD43,285 [SD 11,993] versus USD42,920 [SD 13,931]) were similar for both groups. The results were also similar after weighting.

**Conclusions:**

Patients in Taiwan who underwent TAVR with BEVs had better survival outcomes than those who received SEVs, while other clinical and cost outcomes were comparable between the valve types.

